# WHO consultation on Respiratory Syncytial Virus Vaccine Development Report from a World Health Organization Meeting held on 23–24 March 2015

**DOI:** 10.1016/j.vaccine.2015.05.093

**Published:** 2015-06-20

**Authors:** Kayvon Modjarrad, Birgitte Giersing, David C. Kaslow, Peter G. Smith, Vasee S. Moorthy

**Affiliations:** a Initiative for Vaccine Research, World Health Organization, CH-1211 Geneva 27, Switzerland; b U.S Military HIV Research Program, Walter Reed Army Institute of Research, Silver Spring, MD 20910, USA; c PATH, Seattle, WA 98109, USA; d London School of Hygiene and Tropical Medicine, London WC1E7HT, UK

**Keywords:** Respiratory syncytial virus, Vaccine, Clinical development

## Abstract

Respiratory syncytial virus (RSV) is a globally prevalent cause of lower respiratory infection in neonates and infants. Despite its disease burden, a safe and effective RSV vaccine has remained elusive. In recent years, improved understanding of RSV biology and innovations in immunogen design has resulted in the advancement of multiple vaccine candidates into the clinical development pipeline. Given the growing number of vaccines in clinical trials, the rapid pace at which they are being tested, and the likelihood that an RSV vaccine will reach the commercial market in the next 5–10 years, consensus and guidance on clinical development pathways and licensure routes are needed now, before large-scale efficacy trials commence. In pursuit of this aim, the World Health Organization convened the first RSV vaccine consultation in 15 years on the 23rd and 24th of March, 2015 in Geneva, Switzerland. The meeting’s primary objective was to provide guidance on clinical endpoints and development pathways for vaccine trials with a focus on considerations of low- and middle-income countries. Meeting participants reached consensus on candidate case definitions for RSV disease, considerations for clinical efficacy endpoints, and the clinical development pathway for active and passive immunization trials in maternal and pediatric populations. The strategic focus of this meeting was on the development of high quality, safe and efficacious RSV preventive interventions for global use and included: (1) maternal/passive immunization to prevent RSV disease in infants less than 6 months; (2) pediatric immunization to prevent RSV disease in infants and young children once protection afforded by maternal immunization wanes.

## 1 Introduction and objectives

Dr. Vasee Moorthy (WHO) opened the meeting with a description of key processes that lead to licensure, policy recommendation, prequalification and financing of new vaccines for use in low- and middle-income countries (LMICs). The WHO plays an important role in setting up international standards for the quality, safety and efficacy of vaccines, developing policy recommendations, publishing position papers, and assessing priority vaccines for the United Nations Prequalification Program. The Prequalification Program is managed by the WHO and, in close cooperation with national regulatory agencies and partner organizations, aims to make quality vaccines of priority available for the benefit of those in need. Primary considerations for WHO pre-qualification and subsequent investment by the Global Alliance for Vaccines and Immunization (GAVI) include demonstrated efficacy, product quality, safety, implementation feasibility, and affordability [1].

The meeting focused on the clinical development of RSV vaccines for use in LMICs, rather than in high-income countries (HICs), which was scheduled for a separate discussion (notably at the US Food and Drug Administration in June 2015). Topics discussed included: (1) provision of guidance on RSV vaccine clinical development pathways to support evidence-based policy recommendations in LMICs; (2) RSV case definitions and vaccine efficacy endpoints; (3) priority areas and knowledge gaps that need to be addressed for defining a roadmap to RSV vaccine licensure. Additional considerations will also have to be given to the specification of target populations (Table 1) (*i.e.* pregnant women, infants, children), improvement of RSV surveillance and disease burden estimates, and standardization in the choice, methodology, and interpretation of laboratory assays to assess immunogenicity and facilitate prioritization of the vaccine candidate pipeline. With these goals and objectives in mind, there was a program of presentations and guided discussions that involved representatives from academia, industry, and regulatory authorities.

## 2 Overview of RSV and vaccine development strategies

Dr. Barney Graham (US National Institutes of Health (NIH)) opened with a review of RSV pathogenesis following natural infection and the potential mechanism of disease enhancement observed in the formalin-inactivated RSV (FI-RSV) investigational vaccine trials of the 1960s. The natural history of RSV disease follows from virus tropism for the ciliated epithelia of small bronchioles and type I pneumocytes of the alveoli [2]. The subsequent immune response results in the accumulation of mucus, sloughed epithelium and lymphoid aggregates that obstruct the bronchioles, which partially explains why infants – who have narrower and higher resistance small airways – are more prone to severe bronchiolitis [3]. The mechanism by which the FI-RSV vaccine caused enhanced disease and death, however, differed from the immune responses and pathology associated with natural infection; as the FI-RSV investigational vaccine induced a high titer of binding antibodies relative to the titer of functional inhibiting activity induced, resulting in immune complex deposition and complement activation [4–9]. Additionally, FI-RSV appeared to shift CD4^+^ T-cell immunity to a Th_2_ profile characteristic of allergic inflammation [10,11]. Recent data have demonstrated that the fusion glycoprotein (F) exists in both the native pre-fusion and post-fusion conformations and that virions over time, especially when heated, lose pre-fusion F to an irreversible conformational change to the post-fusion state (*B. Graham, personal communication*). Therefore, the processed virions lose the neutralization-sensitive epitopes present on pre-fusion F and would elicit more post-fusion F-specific antibodies with lower neutralizing potency than observed after natural infection [12,13].

The design of an RSV vaccine that is both safe and effective will have to obviate the mechanism by which FI-RSV caused harm in the past. Before embarking on human trials, the immuno-genicity and safety of vaccine candidates should be assessed in one or more animal models, including those that exhibit FI-RSV-associated immunopathology. Mice are the most facile model for documenting T cell response patterns following infection or vaccination, manifesting illness and weight loss following high-titer infection, and demonstrating lung pathology consistent with vaccine-enhanced illness seen in children, particularly eosinophilia and alveolitis. Although cotton rats do not exhibit any signs of illness, they are more permissive to infection than mice are (especially neonates) and have more delayed viral clearance. Rats have better standardization than mice for pathologic scoring of alveolitis following FI-RSV immunization and viral challenge [14–16]. The bovine model, though logistically more challenging and expensive to work with, is the most analogous to RSV pathogenesis in humans. The challenge of calves with bovine RSV may cause nearly identical pathology in the bronchiolar epithelium, as is observed following natural infection with human RSV in infants [17]. Although bovine RSV has only partial homology with human RSV, the ectodomain of F is 90% identical and neutralizing antibodies to human RSV F can cross-neutralize bovine RSV, allowing indirect testing of human vaccines. Given the safety concerns of previous vaccine candidates, evaluation of RSV vaccine candidates intended for use in antigen-naïve infants will need to be performed in animal models to support a rationale for why the vaccine approach would have an acceptable safety profile in this population.

**Fig. 1:**
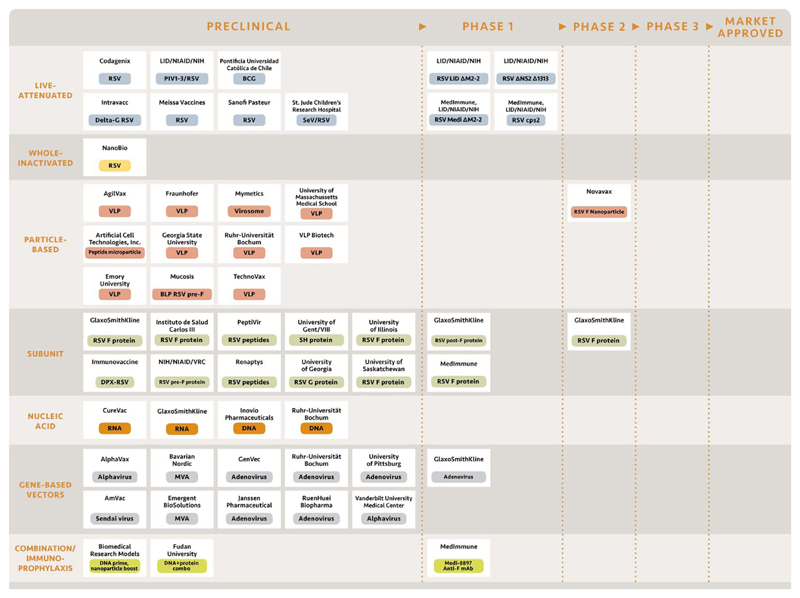
RSV vaccine candidates in pre-clinical and clinical development (adapted from PATH RSV Vaccine Snapshot).

Dr. Ruth Karron (Johns Hopkins University) reviewed promising vaccine candidates entering, or already in, clinical trials. These include more than ten candidates delivered as protein subunits, live-attenuated viruses, or recombinant viral vectors (Fig. 1). The primary goal of RSV vaccination is protection against RSV lower respiratory tract illness (LRTI) in the target population, as induction of sterilizing immunity is unlikely to occur. As infants are the priority population for both active and passive immunization, consideration must be given to how maternal factors may influence vaccine efficacy. These factors include the phenomena of infant immune response suppression by maternal antibody and transplacental antibody transfer in the setting of HIV infection, hypergammaglobulinemia, or placental malaria [18,19]. Developers and regulators will also have to decide whether the guidance used for the past few decades still applies: that only live-attenuated viruses be used in pediatric populations and subunits or other non-replicating vaccines are best used for maternal immunization.

Dr. Peter Collins (NIH) provided additional details on the live-attenuated and live-vectored RSV vaccines currently in clinical development. Live vaccines have the advantage of inducing broad humoral and cellular immunity without requiring an adjuvant and are not likely to cause FI-RSV enhanced disease, as they present viral surface glycoproteins in their native conformations [20,21]. However, because these live viruses induce immunity through replication, they must be highly attenuated [22]. Dr. Collins presented vaccines based on three live attenuated RSV strains, two of which have recently completed phase I clinical trials in RSV seronegative infants. Surveillance data following administration of one candidate suggests that immunization primes for an anamnestic RSV neutralizing antibody response. More than one live-attenuated vaccine candidate is likely to be advanced further for clinical testing in larger and more diverse populations.

## 3 RSV epidemiology: Burden estimates and knowledge gaps

Drs. Janet Englund (University of Washington), Harish Nair (Centre for Population Health Sciences, University of Edinburgh) and James Nokes (KEMRI Wellcome Trust/Warwick University) each presented on the progress and challenges in measuring RSV incidence, disease burden, and mortality in LMICs. One complicating factor is the variation in seasonality of disease burden within and across global regions. While mid-winter epidemics tend to occur in temperate zones, seasonality is less pronounced and occasionally absent in tropical and arctic climates [23–25]. These findings come with the caveat that data from LMICs, particularly in infants less than six months old, are sparse [26] and may require special considerations for additional factors such as low birth weight and ambient air quality. Increasing amounts of RSV hospitalization data are becoming available through influenza surveillance activities, though case definitions may need to be modified to have sufficient sensitivity for detection of RSV cases, particularly in young infants. Incidence rates vary widely across studies due to differences in diagnostic methods, viral subtype, and co-infection prevalence. In addition, there are wide variations in the duration of hospitalization among infants with RSV in different socio-economic settings [27–29].

In 2010, there were an estimated 33 million global cases of RSV-associated LRTI [26]. Although, this estimate was based on community-based studies with active data, it was only from 24 data points. Revised incidence estimates of severe RSV ALRI, based on 73 data points, are currently being calculated (*Harish Nair, personal communication*). Dr. Nokes presented data from one community-based cohort study in Kilifi, Kenya [28,30], which used active surveillance and set criteria for hospital referral as high respiratory rate for age, as assessed by field workers during weekly home visits. The incidence rate of RSV-associated LRTI was six-fold higher when measured by active, compared to passive, surveillance [28].

These findings suggest the incidence or duration of hospitalization due to RSV, used as a primary endpoint in RSV vaccine trials and/or as a surrogate measure of severe disease would be highly variable between settings for reasons unrelated to RSV epidemiology. Furthermore, because many cases of RSV disease do not present to the hospital, there was general consensus on a need for studies involving increased active surveillance or facilitated passive surveillance, linked to community-based data collection, to better inform trial design in LMICs. In addition, background rates of potential adverse events need to be characterized in areas where clinical trials of maternal vaccination are planned for intrauterine fetal demise, congenital malformation, prematurity and intrauterine growth retardation. For trials of live attenuated pediatric vaccines, wheezing in infants should also be monitored. Preparation for pivotal vaccine trials will require a preparatory phase of data collection through longitudinal, epidemiological studies with standardized case ascertainment. This will better inform trial design and result in more robust sample size estimates.

## 4 RSV vaccines in advanced clinical development

Representatives from industry presented the profiles of their most advanced RSV vaccine candidates and discussed target populations, clinical endpoints, trial designs, and safety measures. Dr. Allison August (Novavax) outlined the characteristics of the rosetted post-fusion subunit vaccine that has been shown, in phase II trials, to elicit antibodies that inhibit Palivizumab binding in non-pregnant women of child-bearing age [31,32]. A phase II study to assess safety and immunogenicity in pregnant women is underway and a phase III trial of this vaccine candidate in pregnant women is planned to start in the final quarter of 2015. In this trial, women will be administered a single dose of the vaccine during the third trimester of pregnancy, and their infants will be evaluated for incidence of RSV-associated LRTI with hypoxemia (the decision is still to be made on the oxygen saturation (SpO_2_) threshold) through the first six months of life. The minimal criteria for efficacy and duration of protection were stated to be 60% and 3 months, respectively.

Dr. Filip Dubovsky (MedImmune) described the company’s live-attenuated and live-vectored RSV vaccine program and gave an update on the development [33–35] of their extended half-life monoclonal antibody (MEDI8897), directed at the recently characterized antigenic site Ø on pre-fusion F [12,13]. The live-attenuated vaccines demonstrated shedding, generated a moderate level of antibody responses, and were not associated with enhanced disease. However, increased rates of LRTI that were observed among some vaccinees will require additional evaluation to understand if this finding represents a true safety signal. As for prior vaccine candidates, the efficacy trial endpoints for MEDI8897 will include RSV-associated LRTI.

Although at an earlier stage of clinical development, passive pro-phylaxis with the next-generation monoclonal MEDI8897 appears significantly superior to Palivizumab (a licensed monoclonal antibody for the reduction of serious LRTI caused by RSV infection in high risk infants), with a 9-fold increase in *in vivo* potency and an extended half-life that could offer protection for several months following a single fixed-dose intramuscular administration. Given this potential for greater efficacy, and planned tiered-pricing of the product, a single birth dose of MEDI8897 may ultimately prove cost-effective for protection of infants in LMICs. However, the pathway to prequalification for such a product would need to be created *de novo*, as no monoclonal antibodies are currently prequalified by the WHO.

Dr. Ilse Dieussaert (GlaxoSmithKline Biologicals) described two parallel vaccine development pipelines for maternal and pediatric populations. Phase I data on an adjuvanted recombinant protein subunit intended for maternal vaccination showed no safety signals and moderate immunogenicity with higher neutralizing responses than previous post-fusion F vaccine antigens. As phase III trials are envisioned, particular attention is being given to defining the most reliable and relevant efficacy endpoints for different settings and age groups. Dr. Dieussaert provided a list of signs and symptoms for defining LRTI and severe LRTI that are currently being evaluated in large-scale epidemiologic studies in both high and low resource settings. Although each of the industry representatives proposed a list of possible endpoints for vaccine trials, there was general agreement that RSV-associated LRTI and severe LRTI, however they are to be defined, would be better primary outcome measures than hospitalization (or death).

## 5 Regulatory considerations

Regulators from the US (Dr. Jeff Roberts, FDA Center for Biologics Evaluation and Research), UK (Dr. Mair Powell, Medicine and Healthcare Products Registry), South Africa (Dr. James Southern), and Ghana (Mr. Eric Karikari-Boateng) offered their perspectives on the routes to RSV vaccine licensure. There was general agreement that clinical efficacy studies can feasibly be performed for RSV vaccines and would be required for licensure. In addition to reviewing the quality, safety and efficacy of the submitted product, regulatory authorities will also have to consider specific RSV-related issues. These include the necessity for increased vigilance for vaccine enhanced disease in neonates and antigen-naïve infants, development of a safety database for a first-in-class vaccine to prevent disease in infants through vaccination of pregnant women, and possible use of different vaccine platforms for immunization of pregnant women and young children for the same disease.

In phase III trials, regulatory agencies expect efficacy endpoints to reflect clinically relevant disease prevention, with verification of cases through both laboratory and clinical parameters. Although the minimum number of vaccinees in pre-licensure studies for an adequate safety database is not always prescribed, the numbers required for approval of recently licensed novel vaccines have varied from about 6000 to over 40,000 (the latter in the case of rotavirus vaccines, where theoretical safety signals drove the sample size) [36,37]. The prerequisites for a successful licensure or marketing authorization approval will be addressed on a case-by-case basis in discussion with the manufacturer.

## 6 Duration of follow-up

It was preferred that actively immunized infants should be tracked through two RSV seasons to provide evidence of efficacy, cross-protection against multiple viral strains, and durability of response. While vaccine efficacy is expected to persist for 6 months or less after passive immunization, extended follow-up could be relevant for detection of unexpected adverse events in children who were protected against severe RSV infection during their first season but experienced RSV infection during the second year of life. Deferral of disease may still provide substantial clinical benefit, as older infants are likely to be better able to mount a robust immune response and recover more quickly, with likely lower mortality and fewer long-term sequelae [38,39]. However, it is recommended that the frequency and severity of illness and pattern of immune responses to infection be monitored during the next season. Extended follow up may be considered in the post-marketing surveillance periods, with a specific focus on the impact of immunization on long term wheezing.

## 7 Geographical settings for clinical trials

Clinical efficacy trials of RSV vaccine candidates are likely to be conducted in both HICs and LMICs. Regulators from LMICs emphasized the need for efficacy data relevant to low-resource settings and the importance of defining endpoints relevant to target populations. The oft-used endpoint in HICs of medically attended RSV disease may be less relevant in LMICs. For example, in some settings a significant proportion of children with acute respiratory symptoms may not seek medical care or may make their first clinical contact with a non-medical provider [40]. The choice of primary endpoints in clinical trials will have to take account of the cultural context in which the trials are being conducted. However, it will be desirable to construct widely applicable endpoints with objective clinical criteria to define severe and very severe LRTI, and highly specific validated PCR assays to confirm RSV infection. Collaborations between Northern and Southern hemisphere clinical trials sites and harmonization of clinical endpoints will accelerate the evaluation of vaccines because of the complementary seasonality of RSV infection.

## 8 Clinical development pathway for maternal immunization

As a precaution and a legacy from the experiences of FI-RSV enhanced disease, phase I trials that involve antigen-naïve infants, in any age group or population, should occur in a setting with good facilities for the management of adverse events. These facilities should have the capacity for vigilant follow-up throughout the RSV season and the availability of and access to ventilatory support. For example, the first trials could be conducted in HICs in North America, Europe or Australia followed by trials in lower resource settings. Thus, a staggered development pathway would allow for the procession of trials in lower income settings soon after safety data emerge from higher resource settings.

**Fig. 2:**
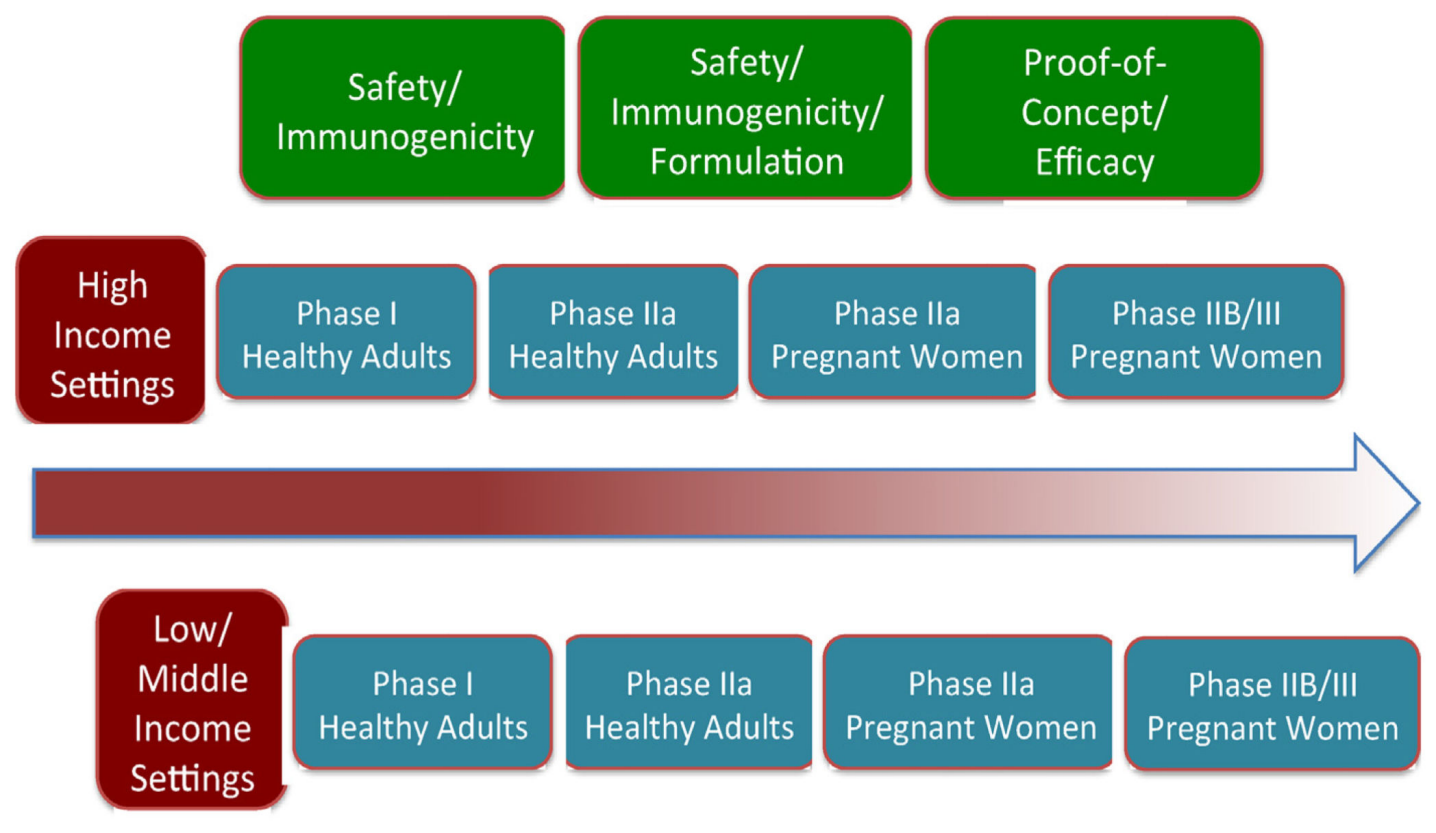
RSV vaccine clinical development pathway for pregnant women.

There was general agreement among meeting participants on the pathway to develop and license an RSV vaccine that would prevent RSV disease in infants less than 6 months of age through maternal immunization (Fig. 2). Novel vaccine candidates that meet preclinical criteria for use in human trials should first be tested in trials that assess safety and immunogenicity in healthy adults, including non-pregnant women. Once data become available from these trials, the dose, schedule and administration route can then be selected from trials in healthy women in their third trimester of pregnancy. Additionally, data will need to be collected on prematurity, intrauterine fetal demise, and other serious adverse perinatal outcomes. A single dose vaccine is desirable, as multiple doses might be associated with decreased uptake. In any trial of pregnant women, both mother and infant should be followed for at least 6 months post-delivery, and preferably for longer into the second RSV season.

One or more preliminary trials in pregnant women may provide sufficient data to demonstrate transfer of functional maternal antibody to the infant, persistence of maternal antibody, and overall reduction of RSV disease in infants, but will not be powered to provide definitive estimates of vaccine efficacy. These preliminary studies will therefore be used to inform the design of one or more larger, confirmatory vaccine efficacy trials. It is also possible that once the dose, schedule and administration route have been selected, preliminary and confirmatory vaccine efficacy data could be obtained from the same trial based on predetermined protocol-specified criteria, *e.g.* by incorporating an event-driven interim analysis. These trials could also evaluate more than one regimen – in the event of continued uncertainty regarding the optimal dose or schedule – by using an adaptive trial design. In this case, emphasis will be placed on the statistical procedures that govern such an adaptive design.

Determination of vaccine efficacy should be based on follow-up of infants for at least 6 months or for as long as maternal antibody has been documented to persist. Trials may need to be carefully timed such that the maternal vaccination period will result in births coinciding with the early part of the RSV season. The follow up in infants for safety is expected to be at least 12 months from delivery, and at least 12 months from vaccination for safety in the mother, and likely longer into the second RSV season. If timed appropriately, it may be possible to conduct more than one confirmatory vaccine efficacy trial across multiple geographical settings. If low and high income settings are merged into a single trial, thought should be given to the design and implementation of case definitions, case detection systems, endpoints, and study procedures that are applicable to all trial settings. Furthermore, the estimated distribution of cases contributing to key endpoints and differing cultural contexts and community engagement procedures must be well understood for each setting prior to trial initiation.

In general, the regulatory approach to the question of benefit (or lack thereof) in pregnant women may be driven by the desired indication sought by the manufacturer. If there is no claim of benefit to pregnant women, then there may be no requirement to demonstrate benefit. For example, the language “prevention of RSV disease in infants through vaccination of pregnant women” does not imply any direct benefit to the mother. However, sponsors are encouraged to collect data on RSV incidence in vaccinated and unvaccinated mothers as is feasible. Co-administration of vaccines is likely to be an important issue as well, particularly in LMICs where fewer antenatal visits mean fewer opportunities to vaccinate pregnant women. In LMICs, tetanus vaccine is likely to be co-administered with a licensed RSV vaccine, while TDaP and influenza vaccines are more likely to be co-administered in HICs.

## 9 Clinical development pathway for pediatric immunization

The approaches to the development of pediatric RSV vaccines are more diverse than those to maternal RSV immunization. For this reason, there was no consensus among meeting participants on a specific framework for this target population. However, meeting participants generally agreed on an initial requirement for studies of safety and immunogenicity in healthy adults. Safety data would be expected from RSV-seropositive subjects before progressing to the target population of seronegative infants. Additionally, it would also be necessary to assess safety and immunogenicity during co-administration with representative routine vaccines administered to target age groups.

## 10 Clinical case definitions for RSV vaccine efficacy trials

Meeting participants agreed that re-analyses of existing epidemiological data and initiation of new epidemiological studies will better inform the design of RSV vaccine trials. After considering case definitions proposed by different groups and ongoing work to update the WHO pneumonia clinical management guidelines, consensus was achieved on candidate case definitions for severe and very severe RSV-associated LRTI (Table 2). The case definitions included clinical features considered to be objective, easily standardized, generalizable across settings, and generally accepted markers of severe or very severe RSV disease. Of note, these case definitions rely heavily upon pulse oximetry. Thus, emphasis was placed on the importance of using appropriate instruments and standardized methods for obtaining pulse oximetry readings. It was proposed that these definitions be piloted in ongoing epidemiologic and surveillance studies, as well as in vaccine efficacy trials. The epidemiological studies could provide valuable information across settings on the sample size needed to demonstrate an effect against severe and very severe RSV-associated LRTI.

## 11 Access for LMIC populations

For vaccine manufacturers the major economic market for RSV vaccines is likely to be in HICs. Post-trial availability of the vaccine in LMICs should be a requirement before RSV vaccine trials are conducted. Stakeholders will have to ask and address the question of when and how is it appropriate to test vaccines in LMICs and what assurances should be in place before such trials occur. In the case of malaria vaccines, it was deemed helpful to include a “neutral party” who would not stand to gain financially if the vaccine was licensed. A product development partnership fulfilled this role for a multi-site African phase III malaria vaccine trial. These are important questions that were not fully addressed at this meeting and merit further evaluation as the RSV vaccine field progresses.

The principle of global access to a safe and effective vaccine has been a well-established principle of previous WHO consultations. Specifically, the WHO will not condone a scenario where a vaccine has been found to be safe and effective partly through testing in LMIC settings but only becomes available in high-income markets. Through the principle of equity, access to vaccines should be based on public health need and not population income. Given that RSV disease burden is disproportionately shifted toward LMICs, there is a major onus on developers/funders to work towards ensuring access, availability and affordability in these settings early in the development and testing cycle.

## 12 Development of reference reagents for RSV vaccines

The majority of RSV vaccine development strategies aim to elicit RSV-specific functional antibodies, as they have long been associated with protection from RSV disease. There are nearly a dozen different assays in use that measure virus neutralizing antibodies, making it difficult to directly compare immunogenicity data across different vaccine candidates. Plaque reduction neutralization (PRNT) is considered the gold standard, but it is a manual, labor-intensive, and lengthy process not easily standardized across laboratories. Microneutralization assays offer some improvement in efficiency through higher throughput detection of viral infectivity. The addition of complement or the use of reporter viruses can also increase assay sensitivity. Still, there is little consensus within the RSV field on what assays, and, more specifically, which method to use and how to report results. Dr. Deborah Higgins (PATH) described an effort by PATH, WHO, and the National Institute for Biological Standards and Control to harmonize data across various formats through the development of a series of clinical assay reference reagents – available to product developers – to facilitate evaluation and enable prioritization of early stage vaccine candidates. The longer-term goal of this activity is to establish one or more of these reagents as International Standards that are applicable to a broad range of assays, enabling comparison of data across studies, regardless of specific assay methodology.

## 13 The concept of an RSV vaccine roadmap

Dr. David Kaslow (PATH) outlined the critical role of the malaria vaccine technology roadmap in prioritizing activities for research, product development, capacity building, policy and commercialization for the purpose of achieving licensure, recommendation and uptake of malaria vaccines. This process has been and continues to be instrumental in establishing a shared vision and strategic goals through consultation with multiple stakeholders, and is reviewed every 5 years or sooner if new data become available that change strategic thinking. The WHO proposed that a similar process be established to identify gaps in the product development pathway for RSV vaccines, to meet the two agreed strategic goals, namely a vaccine for maternal/passive immunization to prevent RSV disease in those under 6 months, and a vaccine for pediatric immunization to prevent RSV disease in infants and young children. This will be drafted through consultation of an RSV Roadmap Working Group, will provide guidance rather than a prescription of the way forward, and is anticipated to be available by mid-2016.

## 14 Conclusions

There are about ten RSV vaccine candidates currently in clinical trials and several dozen in pre-clinical development (Fig. 2). After several decades of addressing major challenges in vaccine design and development, the RSV vaccine field is poised to enter a new phase involving late stage testing of more than one vaccine approach. As RSV disease burden and mortality disproportionately affect infants and young children living in LMICs, actions need to be taken now to ensure pivotal phase III efficacy trials include key populations and endpoints that are relevant to developing countries. An initial step toward clinical development of RSV vaccines for global use was achieved through this WHO consultation. Representatives from higher and lower income countries (Table 3) convened and agreed upon two target populations for vaccine testing and use (pregnant women and young children), the general principles of a clinical development pathway for these two populations (Fig. 2), and candidate case definitions for severe and very severe RSV disease (Table 2).

As more vaccine candidates enter clinical development and efficacy trials, it will be the task of regulators, researchers, manufacturers, and governmental bodies to further refine the agreements and definitions that were discussed at this meeting and to develop population-specific information to optimize vaccine safety, efficacy, and implementation feasibility. To provide guidance toward those ends, the WHO is creating working groups to develop a preferred product characteristics document (to guide target product profiles) and a vaccine roadmap. These guides will offer a more detailed vision of the path forward for an RSV vaccine that is intended for global use.

## Figures and Tables

**Table 1:** Strategic goals for RSV vaccines with a focus on global use.

RSV vaccines for maternal/passive immunization to prevent RSV disease in infants less than 6 months of age
RSV vaccines for pediatric immunization to prevent RSV disease in infants and young children once protection afforded by maternal immunization wanes

**Table 2:** WHO candidate case definitions for severe and very severe RSV associated lower respiratory tract infection (LRTI).

Severe RSV LRTI	Very severe RSV LRTI
An infant or young child presenting to a health facility that is part of the case ascertainment system for the phase III trial who fulfills both the laboratory AND clinical criteria below:	An infant or young child presenting to a health facility that is part of the case ascertainment system for the phase III trial who fulfills both the laboratory AND clinical criteria below:
**Laboratory criterion**	**Laboratory criterion**
RSV infection as confirmed by a fit-for-purpose, fully validated PCR assay with high specificity and sufficient sensitivity on upper respiratory samples	RSV infection as confirmed by a fit-for-purpose, fully validated PCR assay with high specificity and sufficient sensitivity on upper respiratory samples
**Clinical criteria**	**Clinical criteria**
Respiratory infection defined as cough or difficulty breathing	Respiratory infection defined as cough or difficulty breathing
AND	AND
LRTI defined as fast breathing by WHO criteria or SpO_2_ < 95%	LRTI defined as fast breathing by WHO criteria OR SpO_2_ < 95%
AND	AND
≥1 of the following features of severe disease	≥1 of the following features of very severe disease
– Pulse oximetry < 93%	– Pulse oximetry < 90%
– Lower chest wall in-drawing	– Inability to feed
	– Failure to respond/unconscious

**Table 3:** List of consultation participants.

Name	Organization	Location
Participants		
Narendra Kumar Arora	The INCLEN Trust International	New Delhi, India
Louis Bont	University Medical Center, Utrecht	Utrecht, The Netherlands
Harry Campbell	Centre for Global Health Research	Edinburgh, UK
Peter Collins	National Institutes of Health	Bethesda, MD
Janet Englund	University of Washington	Seattle, WA
Barney S. Graham	National Institutes of Health	Bethesda, MD
Eric Karikari-Boateng	Food and Drugs Authority	Accra, Ghana
Ruth Karron	Johns Hopkins Bloomberg School of Public Health	Baltimore, MD
David Kaslow	PATH	Seattle, WA
Shabir A. Madhi	National Institute for Communicable Diseases	Johannesburg, South Africa
Harish Nair	Centre for Global Health Research	Edinburgh, UK
Patricia Njuguna	KEMRI Wellcome Trust	Kilifi, Kenya
James Nokes	KEMRI Wellcome Trust; Warwick University	Kilifi, Kenya; Coventry, UK
Fernando Polack	Fundación INFANT	Buenos Aires, Argentina
Mair Powell	Medicines and Healthcare Products Regulatory Agency	London, UK
Nienke Scheltema	Wilhelmina Children’s Hospital	Utrecht, Netherlands
Claire-Anne Siegrist	Centre Médical Universitaire	Geneva, Switzerland
Eric A.F. Simoes	University of Colorado Health Sciences Center	Denver, CO
Peter Smith	London School of Hygiene and Tropical Medicine	London, UK
James Southern	Medicines Control Council	Simon’s Town, South Africa
Observers		
Allison August	Novavax Inc.	Gaithersburg, MD
Ilse Dieussaert	GlaxoSmithKline Biologicals	Wavre, Belgium
Filip Dubovsky	MedImmune	Gaithersburg, MD
Amy Fix	Novavax Inc.	Gaithersburg, MD
Jorge Flores	PATH	Seattle, WA
Gregory Glenn	Novavax Inc.	Gaithersburg, MD
Pamela Griffin	MedImmune	Gaithersburg, MD
Deborah Higgins	PATH	Seattle, WA
Keith Paul Klugman	The Bill & Melinda Gates Foundation	Seattle, WA
Jean-François Toussaint	GlaxoSmithKline Biologicals	Wavre, Belgium
Niteen Wairagkar	The Bill & Melinda Gates Foundation	Seattle, WA
WHO Secretariat		
Ahmed Bellah	HIS/RSS, WHO-HQ	Geneva, Switzerland
Terry Gail Besselaar	HIP/HSE, WHO-HQ	Geneva, Switzerland
Brigitte Giersing	FWC/IVB, WHO-HQ	Geneva, Switzerland
Ivana Knezevic	HIS/EMP, WHO HQ	Geneva, Switzerland
Kayvon Modjarrad	FWC/IVB, WHO HQ	Geneva, Switzerland
Vasee Moorthy	FWC/IVB, WHO HQ	Geneva, Switzerland
Wenqing Zhang	HIP/HSE, WHO-HQ	Geneva, Switzerland
Tiequn Zhou	HIS/EMP, WHO-HQ	Geneva, Switzerland
